# Erysipelas

**Published:** 1828-08

**Authors:** 


### HOSPITAL REPORTS,

*(Principally condensed from various Periodical Publications.)*

---

### ERYSIPELAS.

*Cases of Erysipelas, treated at the* WESTMINSTER HOSPITAL, *by* MR. GUTHRIE.

I.—JOANNA BURKE, aged thirty, has had several returns of Lichen Syphilitica, and once of Iritis, for which she took mercury until she was in a state of ptyalism. Admitted into the Westminster Hospital with a return of the complaint, and a node on the forehead. On the 8th December, 1827, an incision was made into the node down to the bone, about an inch in length: some matter was discharged, and the bone was bare beneath. This was followed by great relief; but on the 12th she complained of more pain in the head, accompanied by general symptoms of fever, for which she was ordered—

> Hydrarg. Submuriat. gr. v.; Ext. Coloc. c. gr. v. statim; with a cathartic draught in the evening; and Liq. Ammon. Acet. f℥ss.; Liq. Ant. Tart. gtt. xx.; Aquæ f℥j. every four hours afterwards.

13th.—The erysipelatous inflammation of the face became fully developed towards evening; and on the 14th, sixteen ounces of blood were taken from the arm.

> ʒij. of the Sulphate of Magnesia were added to the draughts, with the Mist. Camphor; and fomentations to the face.

On the 15th and 16th, the erysipelas did not increase; and on the 17th she was decidedly better, the pulse still continuing about 100, tongue foul, skin hot, and some headache.

21st.—The erysipelas disappearing, but has still pain in the head and below the left ear, of which she is deaf. Bowels have been continually kept free.

23d.—The Sulphate of Quinine three times a day. The erysipelas entirely gone.

On the 4th January, the Lichen Syphilitica again appeared, and was removed for the fourth time by a course of mercury; which had before caused the first to disappear, but seemed to have little influence on the second, and was not given for the third. She remained well when last seen, the end of May.

II.—Diggory Gordge, aged nineteen, was seized, on the night of the 15th and 16th of June, with erysipelas of the face, accompanied by headache, sickness, a furred tongue, pulse 110, skin hot; the whole face was swelled and red, particularly the nose. He had lately come from the country, and had been admitted into the Westminster Hospital.

He was directed to be bled to f℥ xij.; and to take six grains of the Hydr. Subm. and four of Ext. Coloc. c. immediately; and the following draught every four hours: ℞. Liq. Ammon. Acet. f℥ss.; Liq. Antim. Tart. fʒ ss.; Aquæ f℥j. fiat haustus.

The pulse, shortly after the abstraction of blood, fell to ninety-three, and he was much relieved by it.

On the 17th, the erysipelatous inflammation had began to diminish; the pulse had fallen to eighty.

The pills to be repeated at night, and the medicine continued.

18th.—Better. The remedies to be repeated.

20th.—Well, with the exception of a slight swelling of the features, which still continues.

The common purgative draught to be given daily.

III.—George Vickers, aged forty, admitted 25th May, 1828, into the Westminster Hospital, suffering from erysipelas of the head and face, the consequence of a wound received on the back of the head eight days before.

26th.—The face is greatly swelled, and the features distended; the tongue very furred; skin hot; pulse 120, small and tremulous; bowels confined. He is sensible, and answers questions readily.

Hot fomentations to the face. Six grains of the Hydr. Subm. immediately, and to be followed by a cathartic draught every two hours until the bowels are open; when he is to have ℞. Moschi, Ammon. Carbon. āā gr. iv.; Sp. Æth. Nitr. fʒss.; Mist. Camph. f℥jss. quartis horis.

27th.—Slept in the night for about five hours, although at times delirious. Bowels open; the swelling of the head and face the same as yesterday; pulse 120, tongue furred, great debility.

The purgative to be repeated, and the medicine to be continued.

28th.—Passed a restless night, during part of which he was delirious. Pulse 120, but fuller; tongue cleaner.

Omit the cathartic draught, and continue the medicine every four hours.

29th.—The erysipelas is extending downwards along the neck and breast; at the upper part of the head it has in some degree subsided. Bowels freely open. The Calomel and Colocynth to be repeated at night; and the Mist. Camph. with the Liq. Ammon. Acet. every four hours, without the stimulants, the pulse being fuller, firmer, and ninety-six in number.

30th.—Passed a restless night. Bowels freely open. The swelling is subsiding on the left side of the head and face, but has increased on the right, and is extending on the breast. Tongue cleaner, and upon the whole is better generally.

31st.—Much the same. Pulse ninety-four.

June 1st.—The face more swelled on the right side; the eyelids closed, from effusion into them: they were punctured, and some thin fluid evacuated.

The purgatives and diaphoretics continued.

2d.—Upon the whole, the man is better: locally, there is matter forming in the right cheek, and the cellular membrane over the temporal muscle is partaking of the disease, giving the feel of a quagmire to the finger.—The abscess in the right cheek opened between the angle of the mouth and the lobe of the ear, and good matter discharged. The eyelids also punctured; and three short incisions made in the neck and breast, and a larger one in the course of the temporal muscle.

℞. Infus. Rosæ ℥viij.; Acid. Sulph. gtt. x.; Quininæ Sulph. gr. xvj. sumat f℥j. tertiis horis.

4th.—Complains of a soreness of the mouth from the mercury.

Gargurisma, Chloruret Sodæ ʒiij. ad ℥viij.—A mutton chop and a pint of porter daily.—The Sulphate of Quinine, and wine.

From this time the patient gradually improved, until the end of the month, when he had a relapse, which was again removed by sharp purgatives and diaphoretics, with a cold lotion. There has been a little loss of skin in the course of the long incision from below the zygoma upwards in the course of the temporal muscle, which was laid bare, and is not yet healed.

July 2d.—Discharged to out-patient.

8th.—Readmitted with anasarca of both legs as high as the knees; great debility; pulse weak and ninety; makes little water.

℞. Infus. Gentian f℥jss.; Potassæ Acetatis ʒss.; Sp. Æth. Nitros. fʒss. fiat haustus ter in die sumendus.—℞. Quininæ Sulph. gr. ij.; Micæ panis q. s. ut f. pilula bis die cupienda.—One pint of porter daily, four ounces of wine. Meat diet.

15th.—Is again nearly well; the swelling in the legs almost gone; the secretion of urine regular; the strength greatly reduced.

Mr. Guthrie made these, and other cases to which he alluded, the subject of a clinical lecture. He first drew attention to the fact, that there were several modes of treating erysipelas diametrically opposite to each other, whether with reference to the general or local method of treatment. Dr. Cullen recommended the antiphlogistic plan, with venesections; Dr. Baillie considered bark a specific: both succeeded; and it naturally led to the inference, which was in fact the truth, that there were several kinds of erysipelas, each dependent on the state of the constitution. The discrepancy also which existed as to the local treatment indicated a difference in the extent, character, and seat of the inflammation itself. Mr. Guthrie referred to the observations made by him on erysipelas in the work on Gun-shot Wounds, in which he said all these circumstances were distinctly stated. In the 48th Number of the London Medical Repository, published some years ago, he had related the history of a case of erysipelas of the head and face, in which bloodletting was carried to a greater extent than in perhaps any other case on record. In three of the four cases lately treated by him, he had recourse to venesection, the antiphlogistic plan generally, with cold lotions. It was in comparison with those he drew their attention to the case of Vickers, treated in a different manner. When this man was admitted, there was great prostration of strength; the pulse was 120, giving a tremulous and indescribable sensation to the finger, indicating a want of power, which bleeding would have greatly increased. In cases of this nature, in which venesection had been resorted to, Mr. Guthrie had seen the symptoms greatly increased, a low muttering delirium brought on, such as occurs in typhous fevers, and the speedy dissolution of the patient the result. For this reason, stimulants were had recourse to for the first three days, until the powers of nature were fully developed, when purgatives and diaphoretics were depended on until matter began to form. The sulphate of quinine, with wine and porter, were then had recourse to, and the patient safely carried through a most formidable malady.

The local effects and treatment of the disease were not less deserving attention. On the left side of the head no matter was formed; on the right side, two different formations of matter took place: the first, in the cheek, was a common abscess, which healed after being opened, without any loss of substance. The second was attended by sloughing of the cellular membrane, rendering an incision through the whole length of this affection necessary. Mr. Guthrie said this case was then deserving observation, being one of the very few recorded in which an abscess, forming and healing like a phlegmonous abscess, took place in erysipelatous inflammation. Another instance of the same kind had occurred last year in a woman with erysipelas of the knee. They were, however, very rare, and he believed he had first drawn a distinction between them and the diffused abscess, complicating the cellular texture and destroying it, in his work already alluded to.

In cases of diffused cellular inflammation, he said incisions of a greater or less length were generally necessary, either to prevent or to cure. Many gentlemen differed in opinion on this point, but it appeared to him this difference was principally in discussion, not in the fact: each selecting for their observations different periods of the disease. For instance, some recommended that leeches should be substituted for an incision, supposing it was the loss of blood that occurred after an incision which caused the relief. This was a mistake. It was the removal of the tension that rendered the important benefit. Bleeding by leeches might, in the first instance, prevent the extension, or the continuance of the inflammation in the cellular tissue; but when it has taken place, so as to give it the firmness and fulness preceding the boggy feel alluded to, he was sure it was too late for leeches to afford the desired relief. An incision, or incisions, were required, of a length proportionate to the extent and situation of the disease, varying from simple punctures with a lancet, as in the swelling of the eyelids, to cuts of several inches in length, where the texture of a whole limb was invaded. It was the stretching of the skin by the swelling underneath which gave rise to the constitutional irritation, delirium, and fever, and led to the death of the patient. The accompanying sloughing of the cellular texture beneath proved ultimately destructive to the loss of the skin; but, before gangrene took place, the patient was usually carried off.

Mr. Guthrie stated his belief that the disease was almost always confined to the cellular texture above the fascia, which never, therefore, need be divided; and, by avoiding it, any danger which might be feared from loss of blood would in general be obviated : the vessels which usually continue to pour out blood being there immediately running on, and connected with, the fascia of the limb. In fact, he said, he never wished the patient to lose but a small quantity of blood, the object not being to abstract blood, but to relieve tension, by giving vent to the fluids contained and depositing in the smaller part.

Mr. Guthrie referred to the case of Thomas Key, treated by him in this manner in the Westminster Hospital, in October 1823, as exemplifying all these points in a remarkable manner; that his observations published with the case in his work on Gun-shot Wounds, (page 104 to 111, third edition,) show, that the merit is due to him of introducing long incisions in phlegmonous erysipelas, whilst at the same time the modus operandi of their action was plainly developed.

